# Three-dimensional finite element analysis of the optimal mechanical design for maximum inward movement of the anterior teeth with clear aligners

**DOI:** 10.1038/s41598-024-63907-x

**Published:** 2024-06-08

**Authors:** Jingcheng Chen, Daoyu Zhu, Mengli Zhao, Zhiheng Cheng, Yan Pan, Xin Liu

**Affiliations:** 1 Hefei Stomatological Hospital, Hefei, 230000 Anhui People’s Republic of China; 2 Wanan Medical College, Wuhu, 241000 Anhui People’s Republic of China; 3 Bengbu Medical College, Wuhu, 241000 Anhui People’s Republic of China; 4 Hefei Dental Clinical College of Anhui Medical University, Hefei, 230000 Anhui People’s Republic of China

**Keywords:** Preclinical research, Motility

## Abstract

This study aims to refine clinical designs within clear aligner therapy, exploring the appropriate ratio of anterior tooth retraction to intrusion under maximum anchorage. Using a three-dimensional finite element model and evaluating 19 load scenarios with first premolar extraction, the research identifies the optimal force angle for anterior tooth retraction as 45 to 55°. For clinical planning, it is recommended to design a retraction of 0.19 mm combined with an intrusion of 0.16 mm to achieve anterior tooth retraction. This investigation is crucial for enhancing understanding of biomechanical principles in clear aligner orthodontics, offering significant insights for effective treatments.

## Introduction

Recent advancements in materials science and orthodontic technology have increased the use of clear aligners significantly since the last century. These aligners, compared to traditional braces, offer enhanced treatment experiences in terms of comfort and aesthetics^1^. Clear aligners are easier to clean due to their removability, though some studies report no significant difference in hygiene effectiveness compared to other orthodontic appliances^2–5^. Digital technology in clear aligner design allows for more precise and controlled tooth movement, outperforming conventional methods. Despite these benefits, clear aligners have limitations, mainly due to their dependence on patient compliance for effectiveness^6^. Research indicates that clear aligners achieve an average tooth movement accuracy of about 56.18%, with mesiodistal movements being the most accurate at 72.33%. Intrusion movements, however, show lower accuracy at 43.28%. Overall, the accuracy rate for tooth movement with clear aligners hovers around 50%, but some studies suggest a lower average of approximately 41%, with a notable decrease in precision for certain movements^7–9^. Additionally, undesired tooth movements, such as anterior teeth elongation, can occur in cases of tooth extractions and significant anterior retraction^10^.

In response to the challenges presented by clear aligners, manufacturers have introduced two key solutions. The first involves using two types of aligners with different material properties: one elastic and the other rigid. This strategy aims to improve the efficiency of tooth movement while reducing unintended shifts. The second solution integrates overcorrection in the aligner design and employs mechanical elements to ensure accurate tooth movements^11^. For instance, in treating Angle Class II patients who have had tooth extractions, it’s now common to apply vertical pressure during anterior teeth retraction to boost movement efficiency^12^. Currently, clinical design strategies for clear aligners depend on both the data from aligner companies and the orthodontist’s clinical expertise. However, the precise biomechanical principles and optimal design for orthodontic forces in this area remain unclear.

The advancement of medical engineering technology has enabled the effective use of three-dimensional finite element analysis in orthodontics. Traditionally, orthodontic treatments relied on clinical experience and two-dimensional imaging. However, three-dimensional finite element analysis, a numerical method, can simulate complex structural behaviors^11^. In orthodontics, it helps create detailed three-dimensional models of teeth and jaws, considering biomechanical properties, tissue characteristics, and boundary conditions. This enables the simulation of tooth movement and skeletal remodeling during treatment^12^. The use of this analysis provides more accurate and comprehensive data, improving the efficiency and predictability of orthodontic treatments. The focus of this study is to determine the optimal clinical design for the balance between anterior tooth retraction and vertical pressure during retraction using clear aligners, using three-dimensional finite element analysis to uncover the biomechanical principles involved.

## Materials and methods

A single adult patient was recruited as the study participant, and the research was conducted following the patient’s informed consent. The inclusion criteria for participant selection were as follows: (1) Alveolar bone in the absence of disease, degenerative changes, or significant bone loss, (2) Angle Class II malocclusion, (3) Normal alveolar bone density, and (4) No presence of teeth with developmental malformations, previous root canal treatment, or crown restorations in the oral cavity.

Cone beam computed tomography (CBCT) images, stored in DICOM (Digital Imaging and Communication in Medicine) format, were sourced from an imaging database^13–15^. These images were imported into Mimics 20.0 (Materialise Company, Belgium), a software developed by Materialise NV for 3D reconstruction and analysis in dentistry and orthodontics. In our study, Mimics 20.0 was used for precise three-dimensional modeling of alveolar bone and teeth. Post thresholding and segmentation, focusing on the infraorbital to alveolar bone region, maxillary and dental models were isolated using mask separation and refined before being saved as Stereolithography (STL) files.

These STL files were then processed in Geomagic Wrap 2021(Raindrop Company, United States), where smoothing and noise reduction were applied to create solid models. We adapted previous research methods, using Boolean operations to create a 0.25 mm periodontal model and a 0.75 mm clear aligner model from an existing aligner^16,17^. Attachments measuring 3 mm × 2 mm × 1 mm were designed for cuspid and posterior areas, and the first premolar was removed in the model. These models were assembled in Solidworks 2020 (Dassault Systèmes Company, United States), a 3D CAD program by Dassault Systèmes, and saved as Standard for the Exchange of Product model data (STP) files. Solidworks 2020’s advanced capabilities allowed us to design and optimize the clear aligner structures for Finite Element Analysis (FEA).

**Figure 1:**
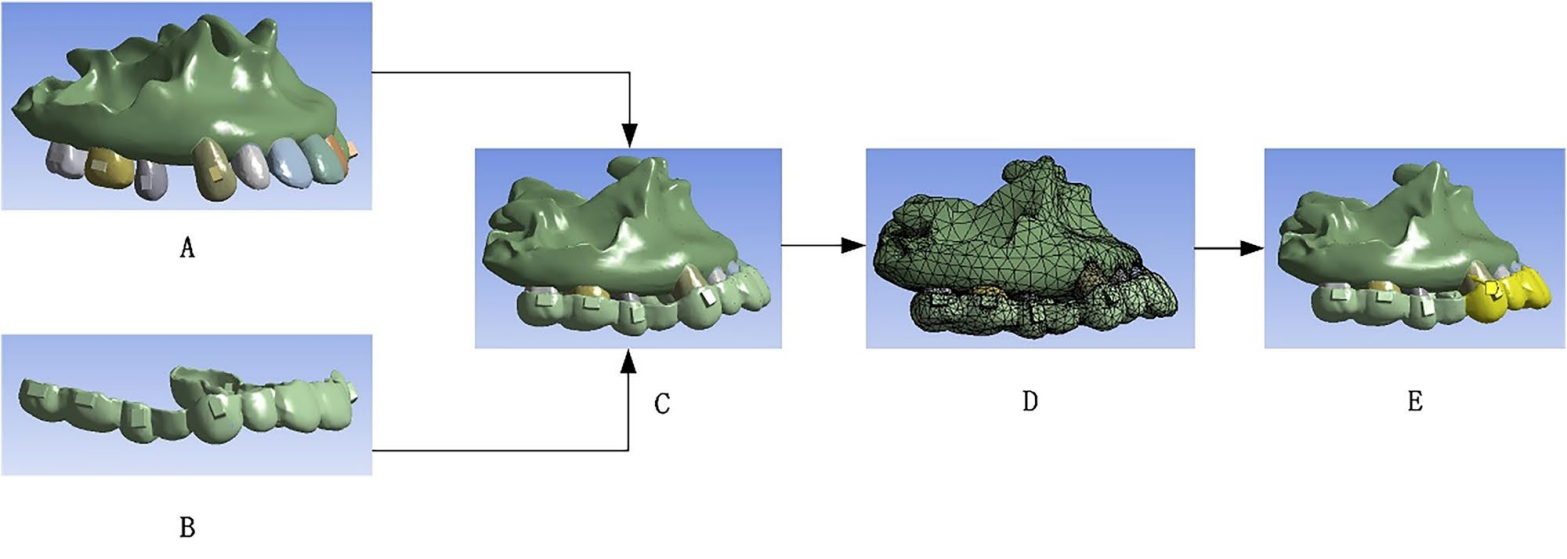
Three-dimensional finite element simulation model. (**A**) Maxillary and dentition model. (**B**) Clear aligner model. (**C**) Overall maxillary model with loaded aligners. (**D**) Meshed model. (**E**) Mechanical loading of the anterior part of the clear aligner.

Finally, Ansys Workbench 2021R1 (Ansys Company, United States), developed by Ansys, Inc., was used to analyze these files. This software is known for its engineering simulation capabilities, and in our study, it was instrumental for FEA to assess mechanical performance and stress distribution of clear aligners on anterior teeth. Ansys Workbench’s analysis provided vital quantitative data on the interaction between teeth and aligners, as shown in Fig. 1.

The parameters such as periodontal membrane, dentition, alveolar bone, clear aligner, attachment elastic modulus, and Poisson’s ratio have been referenced in prior studies. The settings for these parameters are provided in Table 1^13^.

**Table 1:** Material properties.

Materials	Modulus of elasticity (MPa)	Poisson’s ratio
Periodontal ligament	0.69	0.045
Dentition	19,600	0.30
Alveolar bone	1370	0.30
clear aligners	528	0.36
Attachments	12,500	0.36

Meshing was conducted using Ansys Workbench 2021R1. In the finite element (FE) model, the periapical bone was established as a fixed constraint, the interfaces between the dentition and periodontium, periodontium and alveolar bone, and attachment and dentition were set as fixed contacts. Additionally, the relationships between the clear aligner and dentition, as well as between adjacent teeth, were configured as sliding frictional contacts, with a friction coefficient of 0.2 based on prior research^14,15^.

The center point of the maxilla over the mid-palatal suture is designated as the origin of the coordinate system. The X-axis signifies the buccal-palatal coronal axis, with the direction towards the patient’s left side as positive and the direction towards the patient’s right side as negative. The Z-axis represents the vertical axis of the upper and lower structures, with the upward direction as positive and the downward direction as negative. Lastly, the Y-axis corresponds to the labial-palatal sagittal direction, where the palatal side is deemed positive and the labial side is regarded as negative.

**Figure 2:**
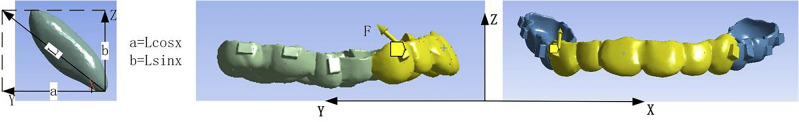
Loading mode of loading. a: Sagittal load, b: Vertical load, L: Maximum design teeth movement per stage of the clear aligner, F: Combined force at the front of the clear aligner, X: Coronal direction, Y: Sagittal direction, Z: Vertical direction.

As illustrated in Table 2, this study simulated loads by replicating the anterior depression and inward displacement of the clear aligner. Drawing on prior research, we formulated a total of 19 load sets in alignment with the clear aligner company’s specification of a maximum movement step of 0.25 mm, considering the force angles on the anterior teeth^9,13,18^. Figure 2 depicts the referenced coordinate system: here, ‘X’ corresponds to the Coronal direction, ‘Y’ signifies the Sagittal direction, and ‘Z’ denotes the Vertical direction. Within this coordinate framework, the parameter L signifies the maximum intended teeth movement per stage, established at 0.25 mm. This value functions as a benchmark for the aligner’s displacement capability. Parameters ‘a’ and ‘b’ denote the anterior inward displacement and anterior depression displacement of the aligner, respectively. These parameters dictate both the magnitude and direction of the aligner’s movement in the anterior region. Additionally, the parameter ‘x’ represents the angle between the force direction and the sagittal direction. By adjusting the ratios of sagittal and intrusion displacement, specific values of ‘x’ can be determined, influencing the overall movement of the maxillary anterior teeth. For detailed information about the particular values of torque and intrusion displacement ratios and their corresponding relationships, please refer to Fig. 2.

**Table 2:** Loading design.

Inward angulation	Sagittal plane loading (mm)	Vertical loading (mm)
0	0.25	0
5	0.249	0.022
10	0.246	0.043
15	0.241	0.065
20	0.235	0.086
25	0.227	0.106
30	0.217	0.125
35	0.208	0.143
40	0.192	0.161
45	0.177	0.177
50	0.161	0.192
55	0.143	0.205
60	0.125	0.217
65	0.106	0.227
70	0.086	0.235
75	0.065	0.242
80	0.043	0.246
85	0.022	0.249
90	0	0.25

### Ethical statement

This experiment has received approval and permission from the Ethics Committee of Hefei Oral Hospital, with the corresponding approval reference number (KQLL2023026). All experiments have been conducted in accordance with relevant guidelines and regulations. Throughout the entire experimental process, for research involving the use of human participant image data, it is confirmed that all studies have been conducted in accordance with the relevant principles and regulations. Furthermore, this study has obtained informed consent from all participants and/or their legal guardians and adheres to the provisions of the Helsinki Declaration.

## Results

**Figure 3:**
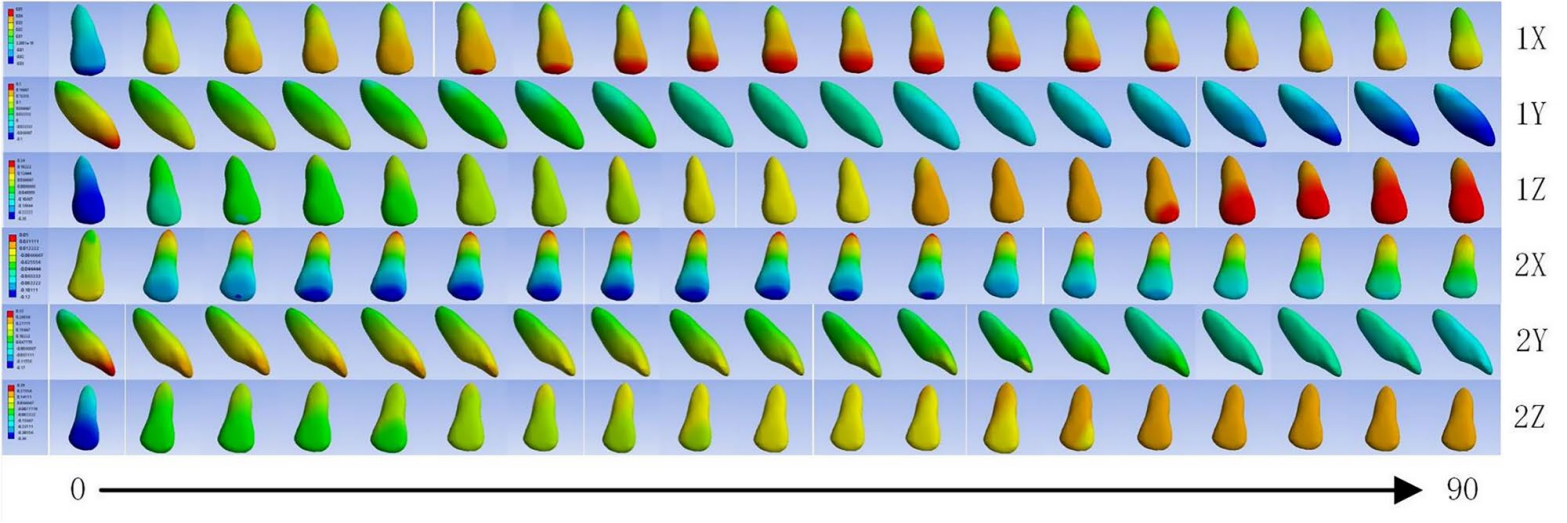
Mechanical results of mesial and lateral incisors under different force angles. 1X: Central incisor coronal direction, 1Y: Central incisor sagittal direction, 1Z: Central incisor vertical direction, 2X: Lateral incisor coronal direction, 2Y: Lateral incisor sagittal direction, 2Z: Lateral incisor vertical direction, 0–90: Representing angles starting from 0° and increasing to 90° according to 5° each time.

Within the context of three-dimensional finite element analysis concerning anterior tooth retraction with clear aligners, the displacement patterns of the crowns and roots of the central incisors and lateral incisors were observed and analyzed (Fig. 3).

### Anterior tooth tip changes

For central incisors, adjusting the anterior force angle of the clear orthodontic aligner to 0° leads to a negative displacement of the crown in the coronal direction. With the force angle incrementally increasing from 5 to 45°, there is a shift in the crown’s displacement towards a positive direction at the tip, peaking at approximately 0.045 mm around an angle of 50°. Past this juncture, the displacement begins to diminish as the force angle moves closer to 90°.

In contrast, the displacement pattern for the crowns of lateral incisors exhibits a distinct behavior. At a 0° force angle, the crown undergoes positive displacement at the tip. However, as the force angle escalates from 5 to 30°, the displacement shifts to a negative direction at the tip, reaching its maximum at about − 0.117 mm around a 35° angle. Following this peak, the negative displacement in the tip gradually decreases as the force angle extends from 40 to 90°, showcasing a unique response in lateral incisors compared to central ones. This differential response highlights the complexity of dental movements and the need for precise control of force angles in orthodontic treatments with clear aligners.

Comparable displacement patterns were noted in the roots of both the central and lateral incisors, closely reflecting the trends observed in their crowns. The roots of the central incisors and lateral incisors demonstrated maximum displacement peaks at an angle of approximately 35°. These peaks measured about 0.017 mm for the central incisors and 0.048 mm for the lateral incisors, respectively. These insights contribute to a detailed understanding of the distinct displacement behaviors and magnitudes for both the crowns and roots of the central and lateral incisors during anterior tooth retraction using transparent aligners.

### Changes in the torque of anterior teeth

In the investigation of anterior tooth retraction utilizing clear aligners, the displacement patterns of the crowns of both central and lateral incisors, as well as the roots of lateral incisors, were meticulously evaluated. Noteworthy findings include:

- For the crowns of central incisors, a distinct polarized displacement pattern in torque was observed. From 0 to 50°, these crowns exhibited positive displacement along the sagittal plane, with a particularly significant displacement occurring at the initial angle shift, especially around 5°. However, as the intrusion force increased, starting from approximately 55°, the displacement direction of the crown in the sagittal plane reversed to negative. This trend culminated at a 90° angle, where the sagittal force was effectively neutralized, leading to a significant reduction in the negative displacement of the crown along the sagittal direction. This shift underscores the nuanced impact of force angle adjustments on tooth movement dynamics, highlighting the complexity of achieving desired orthodontic outcomes with clear aligners.

For the crowns of lateral incisors, there was a noted gradual decline in positive displacement along the sagittal direction, observed from 0 to 85°. Initially, at a 0° angle, the peak positive displacement in torque was recorded at 0.319 mm. As the coronal force was reduced to zero, there was a noticeable shift in the displacement pattern of the crown, transitioning towards a negative displacement in torque. Regarding the displacement of roots, the central incisor root exhibited a peak positive displacement of approximately 0.194 mm in the sagittal direction at the outset, 0°. Approaching the 60° mark, the direction of displacement started shifting towards positive values in the sagittal direction, indicating a complex interaction between the applied forces and the resulting tooth movement. Conversely, the root of the lateral incisor showed minimal movement at 0° in torque, approximately 0.0001 mm, suggesting a significant resistance to displacement in this initial state. From 0 to 60°, the root experienced a positive displacement in torque. Yet, between 65 and 70°, the movement direction of the root switched to negative. Finally, from 75 to 90°, the displacement in torque again shifted to positive values. This pattern underscores the nuanced and differential response of root movements to varying degrees of force application, illustrating the complexity of effectively managing tooth displacement with orthodontic treatments.

### Changes in the intrusion of anterior teeth

During the process of anterior retraction using clear aligners, the intrusion displacement patterns of both the central and lateral incisor crowns were meticulously observed and analyzed. The results of this research are summarized as follows: Initially, from 0 to 20°, the central incisor crown demonstrated negative displacement in the vertical direction. As the force component in the vertical direction increased, a continuous increase in the crown’s intrusion displacement was observed, ranging from 25° up to 90°. Similarly, the lateral incisor crown exhibited a intrusion displacement pattern akin to that of the central incisor, with a notable shift in the direction of displacement occurring around 30°. In conclusion, it is evident that both the central and lateral incisors initially experienced negative displacement in the intrusion direction. However, with the escalation of the vertical component force, their displacement gradually shifted to positive vertical displacement.

## Discussion

**Figure 4:**
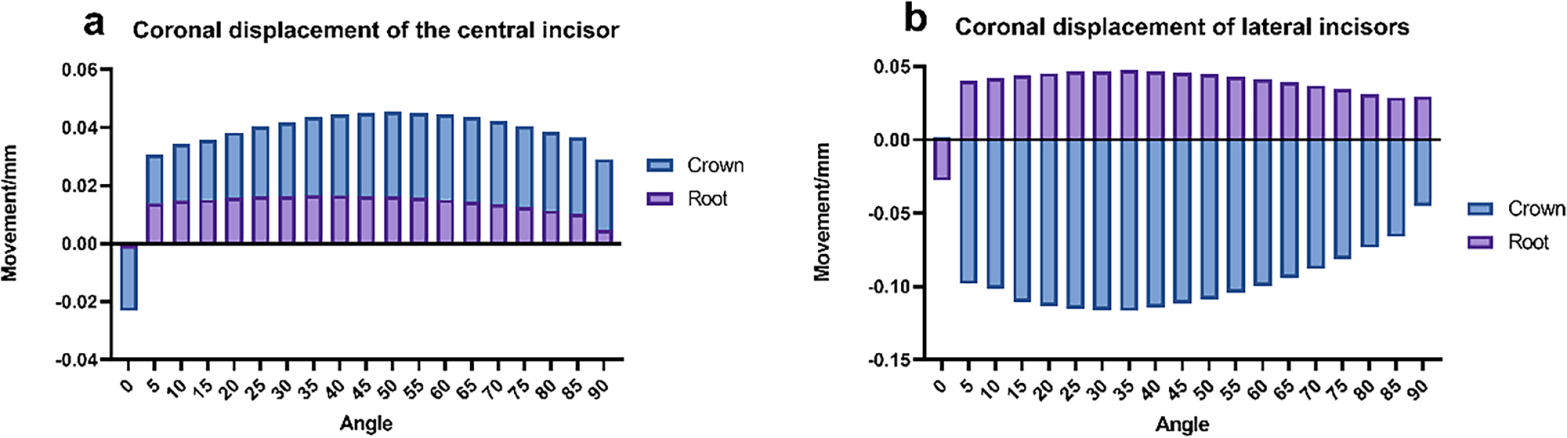
Coronal displacement of mesial and lateral incisors. (**a**) Mesial incisor, (**b**) lateral incisor.

The study demonstrates that variations in the anterior force angle of clear aligners significantly affect the displacement of incisors at the tip, as depicted in Fig. 4. This displacement exhibits variability: during the incisors’ pure retraction phase, the crown undergoes minimal tipping movement. An augmented angle induces increased intrusion force, thereby enhancing the coronal displacement of the crown in a pattern that initially rises before tapering off. Conversely, root displacement remains comparatively negligible throughout this process. Such asymmetric movement between the crown and root leads to irregular modifications at the tips of the incisors, underlining the complexity of orthodontic adjustments with clear aligners.

The behavior of lateral incisors diverges from that of central incisors, showcasing an initial increase followed by a decrease in tip displacement. This pattern, observed consistently across the apical third region, underscores the challenges inherent in managing the root movement of lateral incisors in comparison to central incisors. Sole reliance on vertical compressive forces falls short in effectively controlling the root displacement of lateral incisors. Previous research emphasizes the complexity yet significance of manipulating orthodontic forces using clear aligners, especially on the labial aspect of incisors. The design and strategic placement of attachments play a pivotal role in this context^16^.

**Figure 5:**
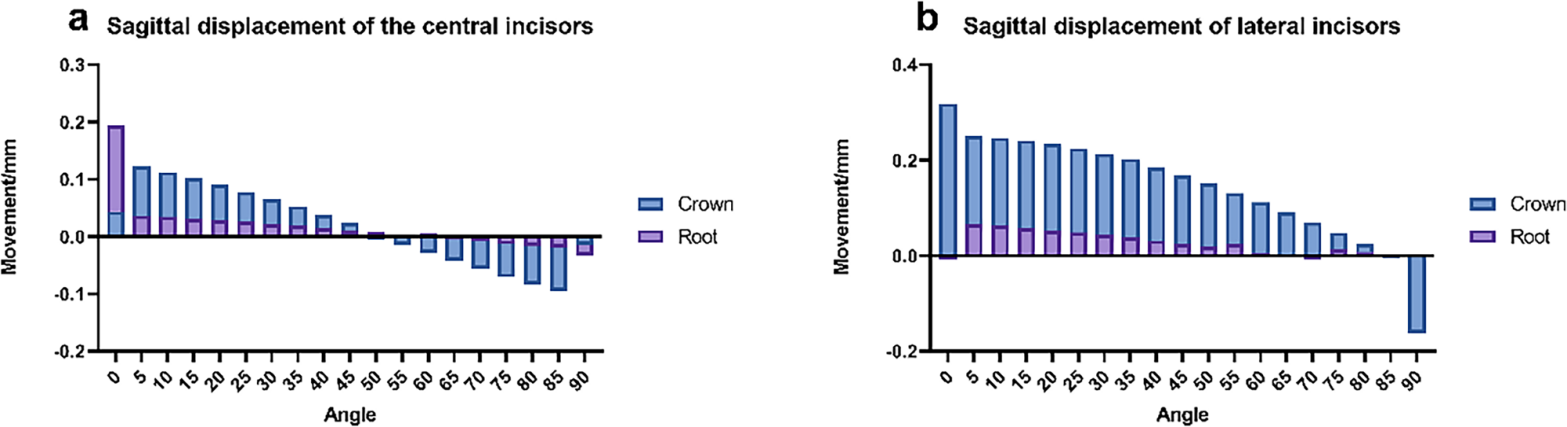
Sagittal displacement of mesial and lateral incisors. (**a**) Mesial incisor, (**b**) lateral incisor.

Optimal tooth displacement occurs in the sagittal plane, yet changes in force angle result in varied coronal and apical displacements, as Fig. 5 illustrates. Excessive sagittal force can cause lingual crown inclination, while too much intrusion force might lead to labial inclination. Comprehensive tooth movement is the objective, but without proper torque control, anterior teeth risk pure palatal tipping and arch effects. Attention to the sensitivity of the center of rotation is vital in anterior teeth translation^17^. Research shows that while the center of resistance’s displacement remains constant regardless of the tooth movement type, different strategies impact alveolar bone remodeling^19^. Precise mechanical distribution in clear aligners is key to accurately targeting the tooth’s center of resistance, ensuring uniform force distribution around the root for comprehensive tooth movement^20^. Therefore, balancing sagittal and vertical forces is essential.

**Figure 6:**
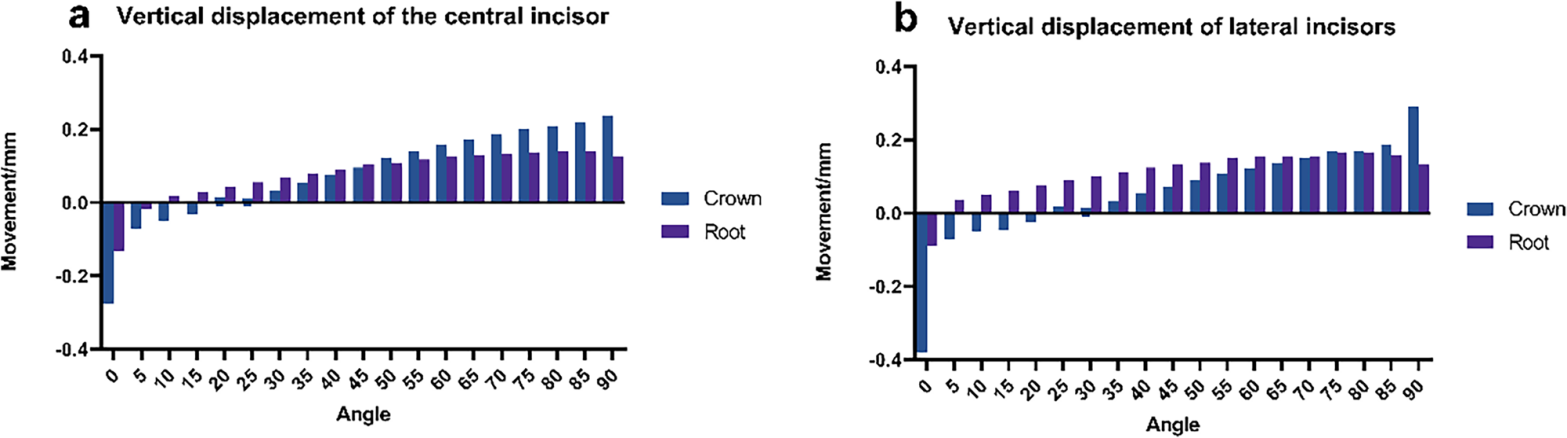
Vertical displacement of mesial and lateral incisors. (**a**) Mesial incisor, (**b**) lateral incisor.

The role of the intrusion force component is vital for the movement of anterior teeth. At lower levels, intrusion forces predominantly impact the root of the tooth; however, as the angle of force escalates, the entire tooth undergoes more pronounced displacement. This effect demonstrates that different parts of the tooth are more sensitive and respond more consistently to changes in force, as illustrated in Fig. 6. Research indicates that both central and lateral incisors undergo significant facial alterations at the tip^21^. The objective is to minimize modifications in the anterior region. Nevertheless, without adjustments to the clear aligner’s design, central incisors will exhibit varied responses to shifts in the anterior force angle.

Three-dimensional finite element analysis, an advanced technique for simulating mechanical changes during orthodontic treatment, emphasizes material properties, contact relationships, structural model layout, and load design. In clear aligners, tooth movement is influenced by initial tooth position, interproximal contacts, load design, and alveolar bone structure^22–24^. Comparing coronal movement disparities in the crown and root of central and lateral incisors reveals that vertical rectangular attachments on canines significantly affect lateral incisors’ tip positions during retraction with clear aligners. This is linked to stress concentration in the canine region, with attachment placement affecting this mechanical state^16^. Research suggests that attachments increase tooth movement inclination and stress, with variations in rotation centers within the dental arch impacting torque^17,25,26^. While vertical rectangular attachments on canines improve incisor movement predictability, they also introduce reactive forces^9^. The anterior force angle’s impact on lateral incisors is relatively marked, showing a segmented function-like trend.

**Figure 7:**
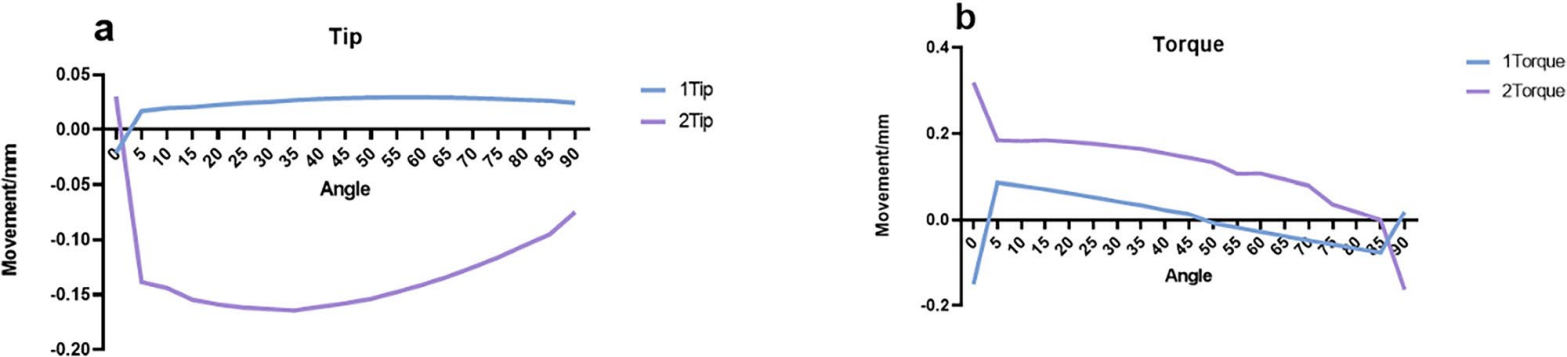
Tip and torque variations. (**a**) Tip, (**b**) torque.

As shown in Fig. 7, the torque trends for both central and lateral incisors are similar, suggesting a reduced impact of canines and their attachments on adjacent teeth in the mesiodistal direction, as opposed to the coronal direction. Central incisors show pronounced torque changes with minimal angle variance between the mesial direction and the Y-axis, attributed to clear aligner force system variations. As the angle increases, central incisors’ coronal and apical movements align, leading to parallel dental arch translation around 50°. Lateral incisors exhibit distinct initial torque changes, which decrease with an increasing angle.

There’s a similarity in displacement trends between central and lateral incisors, but their effects often diverge. Significant torque variation occurs during anterior tooth retraction with clear aligners, particularly without attachments^27^. The three-dimensional displacement of anterior teeth underscores the clear aligner system as a collective mechanical setup, with anterior teeth position and attachments influencing mechanical impacts.

This analysis links the force direction from clear aligners on anterior teeth to their three-dimensional displacements. Central incisor crowns have similar displacements at approximately 35°, while lateral incisors range between 55 and 60°. Anterior teeth roots experience comparable displacement within a 10 to 20° span. However, focusing only on crown and root displacements is inadequate for assessing anterior tooth movement efficiency; tip and torque changes are also crucial for controlling dental roots and axes, and optimizing the labial inclination of anterior teeth.

Comprehensive analysis reveals that below 30°, both lateral incisors’ torque and incisors’ tip changes fluctuate considerably. From 30 to 45°, incisors’ torque variation and tip changes decrease. Between 45 and 55°, incisors’ torque and tip changes are minimal, with limited crown and root displacement. Above 55°, central incisors’ torque decreases, while lateral incisors’ torque rises, especially in intrusion displacement. The optimal force angle range for clinical design is identified as 45 to 55°, correlating with horizontal force direction. Clinical design values in the sagittal direction are between 0.18 to 0.2 mm, and in the vertical direction, between 0.14 and 0.18 mm. These values can be adjusted based on the U1-SN angle and the mandibular plane angle.

Research has found that for patients opting for premolar extraction treatment plans, deformation of clear aligners may lead to unpredictable tooth movement and adversely affect treatment outcomes^28^. Jiang T and their team determined that for clear aligners, a design incorporating 0.2 mm of retraction paired with 0.15 mm of intrusion under moderate anchorage conditions is optimal. However, findings from this study suggest that with maximum anchorage during retraction, it’s possible to decrease the retraction value to 0.18 mm while increasing the recommended intrusion to 0.18 mm. This demonstrates how variations in anchorage design can influence the distribution of mechanical forces on the anterior teeth to a significant degree^13^. Further research has revealed that clear aligners experience specific biomechanical alterations during the gap closing phase, notably the contraction on both sides of the gap and a heightened control of forces. These insights have led to the proposal of more logical, staged design strategies based on the underlying biomechanical mechanisms^29^. Additionally, Gao J and colleagues have identified that moving two molars by merely 0.130 mm can exert the same counteracting force on the anterior teeth as moving a single molar by 0.250 mm. This finding underscores the importance of careful mechanical design for the anterior teeth in cases where posterior anchorage is compromised, to avoid unintended displacement trends^30^.

This study, adhering to the design requirements of clear aligners, devised 19 different clinical movement methods for maximizing anterior tooth retraction under strong posterior anchorage. These designs accounted for the proportional changes between the sagittal and vertical directions and utilized trigonometric functions to calculate the designed load. However, this research is based on a typical model of an Angle’s Class II extraction patient, which has its limitations. The model, with a U1-SN angle of 110°, focuses solely on the maximal retraction of anterior teeth to close gaps. Moreover, to minimize design interference, no attachments were added to the upper anterior teeth model, leaving the interactive effects of attachment and movement designs for future exploration. Additionally, the three-dimensional finite element analysis is based on static mechanical responses, and differences may arise in the actual dynamic mechanics of clear aligners in practice. The clinical effectiveness of the findings from this study requires validation through extensive clinical cases.

## Conclusion

This study employed a three-dimensional finite element technique to analyze anterior tooth retraction across different proportions. The resultant findings can be summarized as follows:

1. Under conditions of maximum anchorage, an optimal retraction angle range of 45 to 55° has been identified. Furthermore, this design can be adjusted based on variations in the U1-SN angle and the mandibular plane angle.
2. For clinical planning, it is recommended to design a retraction of 0.19 mm combined with an intrusion of 0.16 mm to achieve anterior tooth retraction.

## Data Availability

Due to patient information privacy concerns, the datasets generated and/or analyzed during this study are not publicly available. However, they can be obtained from the corresponding author upon reasonable request.

## References

[1] Ke Y, Zhu Y, Zhu M (2019). A comparison of treatment effectiveness between clear aligner and fixed appliance therapies. BMC Oral Health.

[2] Alfawal AMH (2022). The impact of non-extraction orthodontic treatment on oral health-related quality of life: Clear aligners versus fixed appliances—A randomized controlled trial. Eur. J. Orthod..

[3] Ben Gassem AA (2022). Does clear aligner treatment result in different patient perceptions of treatment process and outcomes compared to conventional/traditional fixed appliance treatment: A literature review. Eur. J. Dent..

[4] Borda AF (2020). Outcome assessment of orthodontic clear aligner vs fixed appliance treatment in a teenage population with mild malocclusions. Angle Orthod..

[5] Chhibber A (2018). Which orthodontic appliance is best for oral hygiene? A randomized clinical trial. Am. J. Orthod. Dentofac. Orthop..

[6] Alkadhimi A (2023). Clear aligner orthodontics: What is the evidence for their efficacy?. Prim. Dent. J..

[7] Sachdev S, Tantidhnazet S, Saengfai NN (2021). Accuracy of tooth movement with in-house clear aligners. J. World Fed. Orthod..

[8] Haouili N (2020). Has Invisalign improved? A prospective follow-up study on the efficacy of tooth movement with Invisalign. Am. J. Orthod. Dentofac. Orthop..

[9] Kravitz ND (2009). How well does Invisalign work? A prospective clinical study evaluating the efficacy of tooth movement with Invisalign. Am. J. Orthod. Dentofac. Orthop..

[10] Ren L (2022). The predictability of orthodontic tooth movements through clear aligner among first-premolar extraction patients: A multivariate analysis. Prog. Orthod..

[11] Hong K (2021). Efficient design of a clear aligner attachment to induce bodily tooth movement in orthodontic treatment using finite element analysis. Materials.

[12] Trivedi S (2014). Finite element analysis: A boon to dentistry. J. Oral Biol. Craniofac. Res..

[13] Jiang T (2020). Clear aligners for maxillary anterior en masse retraction: A 3D finite element study. Sci. Rep..

[14] Li Y (2023). Stress and movement trend of lower incisors with different IMPA intruded by clear aligner: A three-dimensional finite element analysis. Prog. Orthod..

[15] Liu L (2021). Effectiveness of an anterior mini-screw in achieving incisor intrusion and palatal root torque for anterior retraction with clear aligners. Angle Orthod..

[16] Takara Y (2022). Mechanical analysis of factors affecting clear aligner removability. Dent. Mater. J..

[17] Cheng Y (2022). Torque movement of the upper anterior teeth using a clear aligner in cases of extraction: A finite element study. Prog. Orthod..

[18] Goto M (2017). A method for evaluation of the effects of attachments in aligner-type orthodontic appliance: Three-dimensional finite element analysis. Orthod. Waves.

[19] Kondo T (2017). Types of tooth movement, bodily or tipping, do not affect the displacement of the tooth’s center of resistance but do affect the alveolar bone resorption. Angle Orthod..

[20] Kuruthukulam RM, Patil AS (2023). The center of resistance of a tooth: A review of the literature. Biophys. Rev..

[21] Mao B (2023). Expansion rebound deformation of clear aligners and its biomechanical influence: A three-dimensional morphologic analysis and finite element analysis study. Angle Orthod..

[22] Verna C (2018). Corticotomy affects both the modus and magnitude of orthodontic tooth movement. Eur. J. Orthod..

[23] Kau CH (2020). 3D analysis of tooth movement using 3D technology. Curr. Osteoporos Rep..

[24] Will LA (2016). Orthodontic tooth movement: A historic prospective. Front. Oral Biol..

[25] Gao J (2023). Biomechanical effects of different staging and attachment designs in maxillary molar distalization with clear aligner: A finite element study. Prog. Orthod..

[26] Cortona A (2020). Clear aligner orthodontic therapy of rotated mandibular round-shaped teeth: A finite element study. Angle Orthod..

[27] Nucera R (2022). Effects of composite attachments on orthodontic clear aligners therapy: A systematic review. Materials.

[28] Mao B (2023). Expansion rebound deformation of clear aligners and its biomechanical influence: A three-dimensional morphologic analysis and finite element analysis study. Angle Orthod..

[29] Wang YG (2024). Dynamic biomechanical changes of clear aligners during extraction space closure: Finite element analysis. Am. J. Orthod. Dentofac. Orthop..

[30] Gao J (2023). Biomechanical effects of different staging and attachment designs in maxillary molar distalization with clear aligner: A finite element study. Prog. Orthod..

